# No Reference, Opinion Unaware Image Quality Assessment by Anomaly Detection

**DOI:** 10.3390/s21030994

**Published:** 2021-02-02

**Authors:** Marco Leonardi, Paolo Napoletano, Raimondo Schettini, Alessandro Rozza

**Affiliations:** 1 Department of Computer Science, Systems and Communications, University of Milano-Bicocca, 20126 Milan, Italy; m.leonardi6@campus.unimib.it (M.L.); raimondo.schettini@unimib.it (R.S.); 2 lastminute.com Group, 6830 Chiasso, Switzerland; alessandro.rozza@lastminute.com

**Keywords:** image quality assessment, Gram matrix, convolutional neural network

## Abstract

We propose an anomaly detection based image quality assessment method which exploits the correlations between feature maps from a pre-trained Convolutional Neural Network (CNN). The proposed method encodes the intra-layer correlation through the Gram matrix and then estimates the quality score combining the average of the correlation and the output from an anomaly detection method. The latter evaluates the degree of abnormality of an image by computing a correlation similarity with respect to a dictionary of pristine images. The effectiveness of the method is tested on different benchmarking datasets (LIVE-itW, KONIQ, and SPAQ).

## 1. Introduction and Background

The advent of digital photography and the growth of the smartphone industry has led to an exponential growth of the number of images taken and stored. The easy access to a camera and the consequent nearly effortless task of taking photos has made shooting a picture tolerated nearly everywhere and at any time. We took photos to capture every moment of our lives, from the simplest one like an unexpected event to milestone events like weddings. Even if cameras and sensors are becoming increasingly sophisticated and precise, it is not rare to shoot pictures that have a low perceived visual quality. Poor external conditions, such as low light environment, backlight scenes, or moving objects, alongside erroneous capturing settings, like exposure, ISO (camera’s sensitivity to light), and aperture, could cause annoying image artifacts and distortions that lead to an unsatisfactory perceived visual quality. Being able to automatically distinguish good quality images from the bad ones can help various types of applications to prune the input set of images, like automatic photo album creation, or even help users to discard bad quality images from their personal collection to save space and time for revisiting those pictures.

Even if the subjective assessment is the most accurate criterion of an image’s quality, it is not possible to perform it over big collections of images in a relatively small amount of time. Without taking into account the expensiveness and the cumbersomeness of manually evaluate pictures, automatic Image Quality Assessment (IQA) algorithms have been widely studied in recent years.

The perceptual quality of an image is a subjective measure and it is usually defined as the mean of the individual ratings of perceived quality assigned by human subjects (Mean Opinion Score—MOS). Given an image, IQA systems are designed to automatically estimate the quality score. Existing IQA methods can be classified into three major categories: full-reference image quality assessment (FR-IQA) algorithms, e.g., [1,2,3,4,5,6,7], reduced-reference image quality assessment (RR-IQA) algorithms, e.g., [8,9,10,11], and no-reference/blind image quality assessment (NR-IQA) algorithms, e.g., [12,13,14,15,16]. FR-IQA methods compare the distorted image with respect to the reference image in order to predict a quality score, and because of that, they requires both the original image alongside the corrupted one. RR-IQA algorithms assess the image quality by exploiting partial information of the corresponding reference image. The quality prediction is the result of a comparison between features extracted from the reference and the image under test. NR-IQA algorithms estimate the image quality solely on the distorted picture without the need of the reference image.

The majority of the existing methods on NR-IQA are designed to be trained on datasets composed of mean opinion scores in addition to the distorted images. Ye et al. in [17] proposed an unsupervised feature learning framework named Codebook Representation for No-Reference Image Assessment (CORNIA). They rely on the use of codebook, a collection of different type of features which encode the quality of an image. The framework can be summed by four major steps: local feature extraction, codebook construction, local feature encoding and feature pooling. In [18], Moorthy et al. present the Distortion Identification-based Image Verity and INtegrity Evaluation index (DIIVINE), a two-stage framework for estimating quality, based on the hypothesis that statistical properties of images change in presence of distortions. These two stages are respectively the distortion identification followed by distortion-specific quality assessment. The core of the method uses a Gaussian scale mixture to model neighboring wavelet coefficients to then extract the statistical description of the distortion in the input image. Lately, most of the works in the state of the arts focus on the use of deep learning: Bianco et al. in [19] propose DeepBIQ, a CNN pretrained on the union of ImageNet [20] and Places [21] datasets fine-tuned for the image quality task. In [22] Celona et al. schematically illustrate building blocks that are implemented and combined in different ways on CNN-based framework for evaluating image quality of consumer photographs.

Nevertheless there are in the state of the art methods which further relax the constraint posed by the previously introduced NR-IQA algorithms, removing the requirement of having human subjective scores, thus leading in two new sub category of the no-reference image quality assessment algorithms namely opinion-aware and opinion-unaware/completely-blind.

Since they do not require training samples of distortions nor of human subjective scores, opinion-unaware methods are usually robust generalization capability. Zhang et al. in [23] propose ILNIQE, an opinion-unaware no-reference image quality assessment algorithm based on natural scene statistics (NSS). In particular, they learn a multivariate Gaussian model of image patches from a collection of pristine natural images. They then compute the overall quality score average pooling the quality of each image patch of a given image using a Bhattacharyya-like distance over the learned multivariate Gaussian model.

In our work, we specifically focus on the no-reference opinion-unaware image quality assessment. Driven by the need of being able to distinguish good quality images from the bad ones, rather than predict a score that best correlate to the mean opinion score, we decide to tackle the problem by an anomaly detection approach. It is well known that convolutional neural networks have the capability of intrinsically encode quality of the images in their deep visual representation [24,25]. In [26], Gatys et al. represent textures by the correlations between feature maps in several layers of a convolutional neural network, and show that across layers the texture representations increasingly capture the statistical properties of natural images. Then in [27] they exploit this correlations between feature maps to create a loss function that measures the difference in style between two images. Other studies reveals how this kind of correlations can be used to classify style of the images [28,29]. In this work, we use for the first time the correlations between feature maps to the task of no-reference opinion-unaware image quality assessment.

The main contributions of this paper are the following:

- We propose an anomaly detection no-reference opinion-unaware image quality assessment by exploiting the correlations between feature maps extracted by means of a convolutional neural network.
- We demonstrate the effectiveness of our method with respect to one no-reference opinion-unaware image quality assessment and two opinion-aware methods, on three databases containing real distorted images.
- We propose a different way to evaluate NR-IQA methods based on their capabilities to discriminate good quality images from the bad ones.
- We show the advantages of combining the correlations between feature maps with an anomaly detection method.

The rest of the paper is organized as follows. In Section 2 we describe in detail the proposed method. Section 3 presents the databases taken into account for the experiments and the metrics used for the evaluation of the proposed method. In Section 4 we report the experimental results, concluding with the Section 5.

## 2. Proposed Method

**Figure 1:**
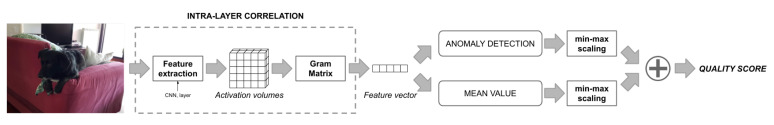
Schematic view of the proposed method. The intra-layer correlation is computed by the Gram matrix over the activation volumes of a Convolutional Neural Network (CNN). Then the *abnormality* and the average of the correlation are computed before applying the min-max scaling on both of them. In the end, the two metrics are summed resulting in the predicted image quality score.

The aim of the proposed method is to estimate the quality score of a given picture exploiting the correlations between the feature maps coming from a VGG16 [30] trained on the Imagenet dataset [20]. In particular, we combine the arithmetic mean of this correlations, and a score resulting from an anomaly detection method, on the intra-layer correlation represented by the Gram matrix. The final image quality score is given by the sum of these two metrics after applying min-max scaling to them. A brief overview of the method is depicted in Figure 1.

### 2.1. Intra-Layer Correlation

The intra-layer correlation has been firstly introduced by Li et al. [26] as representative of the texture, and since then it has been used in several other studies: as a loss [27,31] for style transfer, or to classify the style of the images [28,29]. This property can be defined as the correlation between the feature maps in a given layer and it can be done by calculating the Gram matrix of the feature maps, which is defined as all possible inner products of the feature maps. The Gram matrix has also been used in several other domains and applications: from computing linear independence of a set of vectors to represents kernel functions as in [32].

**Figure 2:**
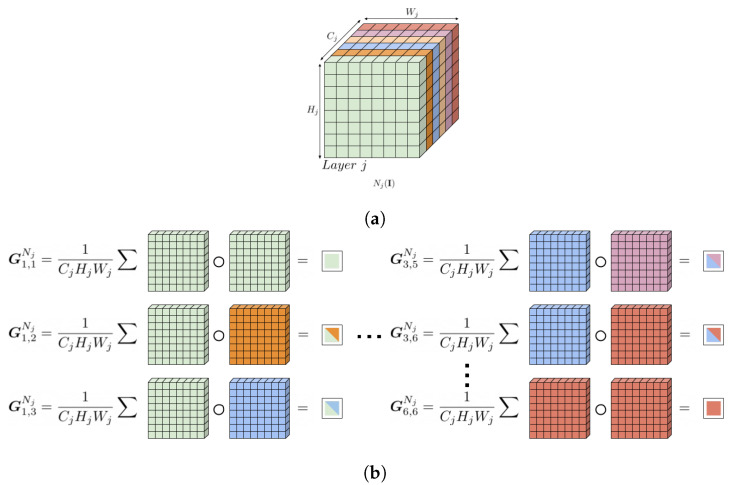
Schematic view of Gram matrix computation. In (**a**) is reported the feature maps of the *j*-th layer. (**b**) illustrate, for some of the indices of the Gram matrix, how they are computed. With the symbol ∘ we refer to the element-wise matrix product.

Given an input image I and a neural network $N_{e}$ made of *e* hidden layers which can be interpreted as the functional composition of *e* functions $L_{j}$ followed by a final mapping $\bar{L}$ that depends on the task: $N_{e}=\bar{L}\circ L_{e}\circ \ldots \circ L_{1}$. Let $N_{j}\left(I\right)$ be the feature maps of the *j*-th layer of the network $N_{e}$ for the input I, with j≤e, which can be seen as a matrix of shape $C_{j}\times H_{j}\times W_{j}$ resulted from the application of the functional composition of the first *j* functions $L_{1},\cdots ,L_{j}$ of the network $N_{e}$ such that $N_{j}=L_{j}\circ \ldots \circ L_{1}$. For the layer *j*-th the Gram matrix $G^{N_{j}}$ can be defined as a matrix of shape $C_{j}\times C_{j}$ whose elements are given by:

(1) $$G_{c,c^{\prime }}^{N_{j}}=\frac{1}{C_{j}H_{j}W_{j}}\sum_{h=1}^{H_{j}}\sum_{w=1}^{W_{j}}N_{j}{\left(I\right)}_{c,h,w}N_{j}{\left(I\right)}_{c^{\prime },h,w}$$

where *c* and $c^{\prime }$ are the indices of the Gram matrix, and both vary in the range $\left[1,C_{j}\right]$. We report in the Figure 2 an illustration of how the Gram matrix is computed from a feature maps $N_{j}\left(I\right)$.

$G^{N_{j}}$ captures which features tend to activate together, therefore we expect $G^{N_{j}}$ to be a symmetric matrix with a negligible diagonal representing the correlation between a filter and itself. For our purpose we can consider only the lower triangular matrix of $G^{N_{j}}$, which once flattened results in a feature vector $x_{j}$ of size $1\times [\left(C_{j}\left(C_{j}+1\right)/2\right)-C_{j}]$. For each layer *j* of the VGG16, in the Table 1 we report the resulting feature vector dimension representing the intra-layer correlation.

**Figure 3:**
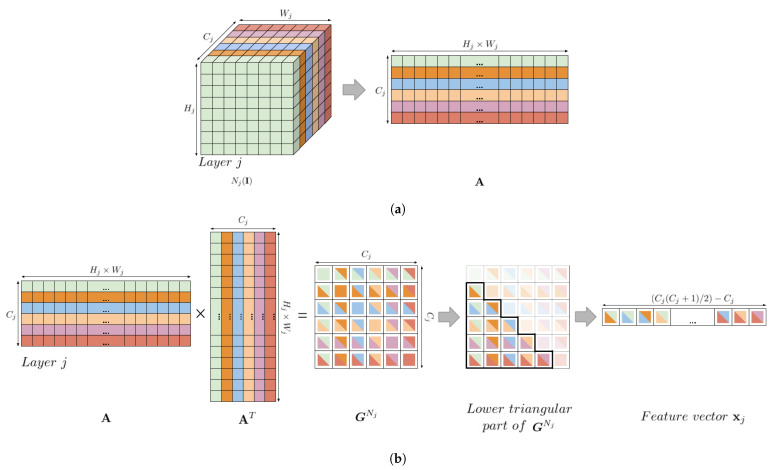
Visual overview of the Gram matrix efficient computation and feature vector extrapolation. In (**a**) is shown how the activation volume is reshaped to efficiently compute the Gram matrix in (**b**) to finally compute the feature vector that represents the intra-layer correlation.

The Gram matrix is invariant with respect to the size of the input image I since its dimension depends only on the number of filter $C_{j}$ of the *j*-th layer, and it can be computed efficiently if $N_{j}\left(I\right)$ is reshaped into a matrix A of size $C_{j}\times \left(H_{j}\times W_{j}\right)$ so that we achieve $G^{N_{j}}=AA^{T}/C_{j}H_{j}W_{j}$. We report in Figure 3a schematic overview of the Gram matrix efficient computation.

### 2.2. Anomaly Detection

Our anomaly detection technique is inspired by the approach presented in [33]. The degree of abnormality, that is the presence of distortions in the input image, is computed by measuring the similarity between the aforementioned intra-layer correlation representation of a given image, and a reference dictionary W of Gram matrices computed from a database of pristine images. The similarity is measured through the Euclidean distances between the feature vector x extracted from a given image I and the feature vectors of the dictionary W. The final *abnormality score* is the sum of the average distances and α times the standard deviation of the distances. It is defined as follow:

(2) $$Abnormality\;score=\frac{1}{D}\sum_{d=1}^{D}dist\left(x,w_{d}\right)+\alpha \sqrt{\frac{1}{D}\sum_{d=1}^{D}\left(dist\left(x,w_{d}\right)-\frac{1}{D}\sum_{d=1}^{D}dist\left(x,w_{d}\right)\right)}$$

where *D* is the number of words in the reference dictionary $W=\{w_{1},\ldots ,w_{D}\}$, and $dist(x,w_{d})$ is the Euclidean distance between the feature vector x representing the input image I and the words of the dictionary $w_{d}$.

The dictionary is built from a subset of pristine images $P=\{I_{1},\ldots ,I_{D}\}$: for each image $I_{d}$ we compute the Gram matrix respect a given *j*th layer of the network $N_{e}$ and keep only the flattened lower triangular matrix of $G^{N_{j}}$ of shape $\left(C_{j}\left(C_{j}+1\right)/2\right)-C_{j}$. We then reduce the dimension of the feature vector to *M* with $M<\left(C_{j}\left(C_{j}+1\right)/2\right)-C_{j}$ by applying the Principal Component Analysis (PCA) [34] to all the feature vector computed from P. *M* represents the number of principal components such that a given percentage of the data variance is retained.

Finally, we group all the reduced features vectors of P into clusters using Mean Shift [35], a centroid-based algorithm which use kernel density estimation to iteratively move the sample to the nearest local maxima of the probability density with respect to the input samples. It only has a parameter named bandwidth which represents the width of the kernel, and the output are the best *k* clusters corresponding to *k* centroids. The advantage of using Mean Shift as clustering algorithm is that it does not require to explicitly define the number of clusters *k* in advance. The number of clusters is instead variable and depends solely on the data and the width of the kernel chosen. A centroid is the most representative point within the cluster, and in this case, is the mean position of all the elements of the cluster. Our dictionary W is therefore composed by the coordinates of the center of each cluster found.

**Figure 4:**
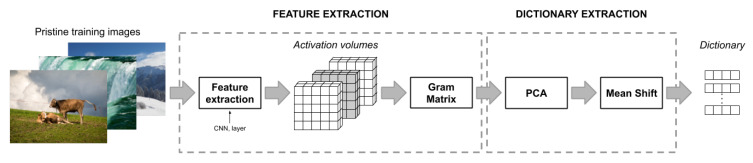
Overview of the creation of the dictionary for the degree of abnormality computation. The activation volumes of a given CNN are extracted and then the Gram matrix is computed to get the intra-layer correlation for a subset of pristine images. Subsequently dimensionality reduction is applied through Principal Component Analysis (PCA). Finally Mean Shift algorithm is performed to compute clusters on which centroids are then extracted as entry of the dictionary.

A schematic view of the dictionary creation pipeline is presented in the Figure 4.

### 2.3. Combining Method

The final quality score is the combination of factors: (1) the arithmetic mean of the CNNs correlations and (2) the *abnormality score*.

To make these two factors comparable we scale them [36]. Moreover, we flip the correlation of the *abnormality score* by computing 1—abnormalityscore. Then, with the purpose of having better interpretability, we divide by 2 and multiply by 100 the final score in order to have values that ideally range between 0 and 100 as the MOS.

Since the anomaly detection method relies on the concept of distance from a dictionary of pristine images, it implies that the closer the input image is to this dictionary, the less abnormal is the considered picture. On the contrary, the farther the input representation is from the dictionary, the higher is the *abnormality score*. The output of the anomaly detection method is therefore negatively correlated with the Mean Opinion Scores.

On the other hand, it is reasonable to believe that a quality artefact in an input image of a CNN, may alter the activation maps, hence it affects negatively the average intra-layer correlation: the more corrupted (less qualitative appealing) is the input image, the less correlated are the layers of the network. It reflects that the average of the intra-layer correlation is positively correlated with the Mean Opinion Scores.

## 3. Experiments

In this section, we start describing the databases taken into account for the experiments, followed by the metrics used for the evaluation of our approach and then the implementation details of the proposed method.

### 3.1. Database for Image Quality Assessment with Real Distorted Images

There are several databases for image quality assessment with real distorted images. For our experiments we take into account one of the most used dataset in the field of IQA which is the LIVE in the Wild Image Quality Challenge Database [37] (LIVE-itW) alongside with two recently presented large scale IQA databases namely: Smartphone Photography Attribute and Quality database (SPAQ) [38] and the KonIQ-10k [39] (KONIQ).

The task of gathering opinion scores on the perceived quality of images is highly subjective and subtle, for all the previously mentioned databases, therefore, every worker was first provided with detailed instructions to help them assimilate the task. Since LIVE-itW and KonIQ-10 rely on crowdsourcing frameworks, a selection of participants based on the worker’s reliability was applied. Also, during and after the process of data annotation several filtering steps were implemented to ensure an acceptable level of quality for the resulting Mean Opinion Scores. To further validate the credibility of the collected scores, the authors of the three databases, also reports the mean inter-group agreement as the mean agreement between the MOS values of non-overlapping random groups of users in terms of Spearman’s rank ordered correlation. In particular, the LIVE-itW database reaches a value of 0.9896 while KonIQ-10 and SPAQ measure a correlation of 0.973 and 0.923 respectively.

The LIVE-itW database [37] is a collection of 1162 authentically distorted images captured from many diverse mobile devices. The images have been evaluated by over 8100 unique human observers through amazon mechanical turk and each image has been viewed and rated on a continuous quality scale by an average of 175 unique subjects. The Mean opinion scores ranges from 0 to 100.

The KONIQ database [39] contains 10,073 images selected from the Yahoo Flickr Creative Commons 100 Million dataset (YFCC100M) [40]. Each image has a total of 120 reliable quality ratings obtained by crowd-sourcing, performed by 1459 subjects. The collected values of the Mean Opinion scores belongs to the interval [0,5], for readability, in this paper, we scale them in the range [0,100] to be coherent with the other datasets.

Finally, the SPAQ database [38] consists of 11,125 pictures taken by 66 smartphones. The images have been labeled through a subjective test, in a well-controlled laboratory environment, with a total o 600 subjects. Each image has been rated at least 15 times. The MOS varies between 0 and 100.

The datasets for image quality assessment with real distorted images are divided into good quality images and bad quality images according to the MOS. As a threshold, for each dataset, we decided to take the 75th percentile with respect to the MOS distribution. Therefore, we label images having a MOS over the 75th percentile as good quality images, while we labeled as poor quality images the remaining ones. These thresholds are 71.71 and 71.74 for the KONIQ and LIVE-itW respectively while for the SPAQ the 75th percentile respect the MOS is 68.82.

**Figure 5:**
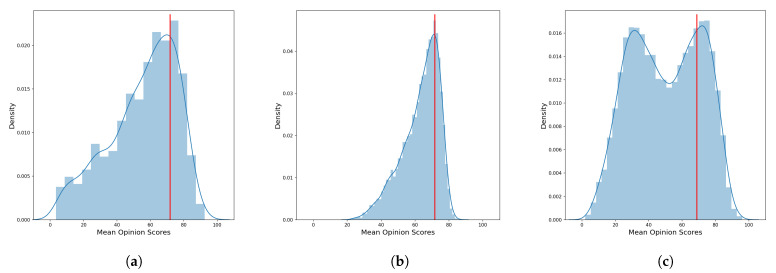
Estimated density distribution of Mean Opinion Scores for the three datasets: (**a**) LIVE in the Wild Image Quality Challenge Database (LIVE-itW), (**b**) KonIQ-10k (KONIQ), and (**c**) Smartphone Photography Attribute and Quality database (SPAQ). The bars represents the normalized histogram, the blue line is the estimated density distribution while in red line is the 75th percentile respect the Mean Opinion Score (MOS).

**Figure 6:**
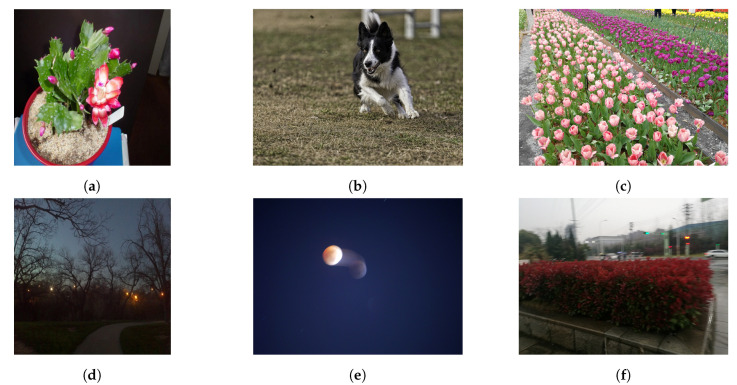
Sample images form the three Image Quality Assessment (IQA) databases: (**a**) and (**d**) images from the LIVE-itW with a MOS of 78.81 and 43.67 respectively, (**b**) and (**e**) KONIQ’s pictures with a MOS of 71.46 and 43.36; finally (**c**) and (**f**) photos form SPAQ whit a MOS of 75.43 and 33.0 respectively.

In the Figure 5 we report the distributions of the Mean Opinion Scores of the three datasets where it can be seen that KONIQ and LIVE-itW seam to distribute similarly with unimodal behaviour, while the SPAQ’s distribution of the MOS appear to be bimodal. An overview of database properties is provided in Table 2 and samples images alongside MOS are in Figure 6.

### 3.2. Database for Image Quality Assessment with Real Synthetic Distortion

**Figure 7:**
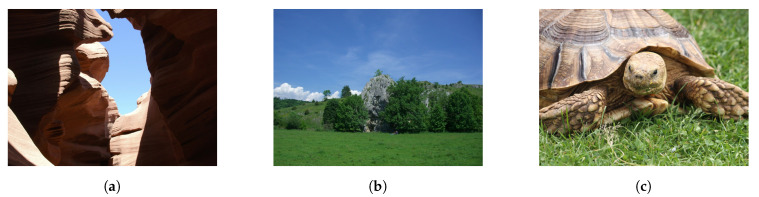
Sample images form the KADIS700k databases. (**a**–**c**) random photos from KADIS700k.

As our pristine collection of images, we decide to take into account a large scale database for image quality assessment with synthetic distortion named Konstanz Artificially Distorted Image quality Set (KADIS700k) [41]. For our purpose, we consider only the non-distorted pictures. The dataset is composed of 140,000 pristine images collected from Pixabay.com, a website for sharing photos and videos. According to the image quality guidelines of Pixabay.com, users must upload pictures that have a well defined subject, clear focus, and compelling colours. Also, images with chromatic aberration, JPEG compression artefacts, image noise, and unintentional blurriness are not accepted. Moreover, authors of KADIS700k have collected up to twenty independent votes by Pixabay users claiming that the quality rating process provides a reasonable indication that the released images are pristine. After selecting 654,706 images with resolutions higher than 1500×1200 they randomly sampled 140,000 images as pristine reference images. Under the aforementioned conditions is our belief that KADIS700k can model the concept of pristine images necessary to build our system. An example of the pictures present in the dataset is reported in Figure 7.

### 3.3. Experimental Setup

The most commonly used metrics to evaluate the performance of no-reference image quality assessment methods are respectively the Pearson’s Linear Correlation Coefficient (PLCC) and the Spearman’s Rank-order Correlation Coefficient (SROCC). Both are used to compare the scores predicted by the models and the subjective opinion scores provided by the dataset. The PLCC correlation evaluates the linear relationship between two continuous variables while the SROCC evaluates the monotonic relationship between two continuous or ordinal variables. Both indexes are used to evaluate correlation between variables. However, according to [42], the Spearman’s index is more suitable to evaluate relationships involving ordinal variables than the Pearson’s index. The aim of the automatic image quality assessment methods is to predict a quality index of the image which simulates the Mean Opinion Score achieved thanks to subjective evaluation of users. Since both the quality index and the MOS are continuous and ordinal variables, the SROCC is the most reliable evaluation metric for IQA methods.

The PLCC is a statistic that captures the linear correlation between the predicted scores and MOS; it ranges between +1 and −1, where a value of +1 or −1 reflects a total positive, or negative respectively, linear correlation, and a value of 0 is no linear correlation. Given *n* as the number of the considered samples, $x_{i}$ and $y_{i}$ the sample points indexed with *i*, $\bar{x}$ and $\bar{y}$ the means of each sample distribution; we can define the PLCC as follows:

(3) $$PLCC=\frac{\sum_{i=1}^{n}\left(x_{i}-\bar{x}\right)\left(y_{i}-\bar{y}\right)}{\sqrt{\sum_{i=1}^{n}{\left(x_{i}-\bar{x}\right)}^{2}}\sqrt{\sum_{i=1}^{n}{\left(y_{i}-\bar{y}\right)}^{2}}}.$$

Instead, the SROCC operates on the rank of the data points ignoring the relative distances between them, hence assesses the monotonic relationships between the actual and predicted scores. As the PLCC, it varies in the interval [+1,−1] and for *n* considered samples it is defined as follow:

(4) $$SROCC=1-\frac{6\sum_{i=1}^{n}d_{i}^{2}}{n\left(n^{2}-1\right)},$$

where $d_{i}=(rank\left(x_{i}\right)-rank\left(y_{i}\right))$ is the difference between the two ranks of each sample.

To measure the capability of the methods of being able to discriminate good quality images from the bad one, we decided to take into account the area under a receiver operating characteristic (ROC) curve, abbreviated as AUC, and the area under the precision-recall curve (AUPR). In particular the AUC measures the overall performance of a binary classifier, and it ranges between 0.5 and 1.0, where the minimum value corresponds to the performance of a random classifier while the maximum value represents the oracle. Meanwhile, the AUPR reflects the trade-off between precision and recall as the threshold varies.

### 3.4. Implementation Details

The proposed method is trained and tested using Python. The training process consists of extracting the information of intra-layer correlation, from a subset of pristine images of KADIS700k [41], and compute the dictionary W of *k* centroids trough Mean Shift. Then the average intra-layer correlation and the degree of abnormality is computed from a different subset of pristine images of KADIS700k [41] in order to save the minimum and maximum value of both to lately perform min-max scaling in order to merge them. The parameter α for the anomaly detection method has been chosen performing a parameter tuning over a subset of synthetically distorted images for KADIS700k, resulting in a value of 2.

For the intra-layer correlation, we use the ImageNet [20] pre-trained VGG16 provided by the Torchvision package of the PyTorch framework [43]. The model was trained scaling the input images into the range of [0, 1] and then normalized using mean [0.485,0.456,0.406] and standard deviation [0.229,0.224,0.225]. As preprocessing images were first cropped such that the resulting size was evenly distributed between 8% and 100% of the original image area and then a random aspect ratio between 3/4 and 4/3 of the original aspect ratio was applied. The resulting crops were finally resized to 224 × 224. As layer for our representation, we rely on the output of the first convolutional layer of the second convolutional stage (conv2_1) of the VGG16, which consists of 128 filters, resulting in a feature vector of shape 8192. Moreover, the input of the VGG16 images are scaled so the smaller edge results of 512 pixels and normalized according to the mean value and standard deviation of ImageNet.

For the training phase, we randomly selected 10,000 pristine images from KADIS700k, and we compute the Gram matrix for each image. Subsequently, we apply the PCA with a percentage of retained variance of 97%. Finally, we use the Mean Shift algorithm with a flat kernel to find the representative centroids and build the dictionary for the computation of the *abnormality score*. The bandwidth for the Mean Shift was empirically estimated as the average pairwise distance between the samples that are in the same cluster applying the Nearest Neighbors algorithm. The resulting bandwidth is 1.804.

To perform the min-max scaling on both the *abnormality score* and the average value of the intra-layer correlation, we randomly select 1000 pristine images from KADIS700k different from the previous ones and we collect the minimum and maximum values for the two metrics.

The proposed method is compared against three different no-reference benchmark algorithms for the IQA, one opinion unaware, which does not require MOS during the training phase named ILNIQE [23] and two opinion aware methods which require the mean opinion scores of the images on which they are trained, namely CORNIA [17] and DIIVINE [18]. For these methods, we took their original implementations alongside the saved models released from the authors. For this reason, these methods are executed a single time.

## 4. Results

In this section, we compare the average performance in terms of SROCC, PLCC, AUC, and AUPR on the three considered databases (LIVE-itW, KONIQ, and SPAQ) across 100 iterations. We first present the average performance across all the three datasets and then we present the performance on each dataset separately.

### 4.1. Average Performance

Table 3 reports the average performance over the three databases of our opinion-unware method and state-of the-art methods, both opnion unware and opinion aware. This table permits to have a quick look of the best performing method whatever is the database considered. Our proposal is on average the first in terms of SROCC, AUC and AUPR against all the methods. In terms of PLCC, our method is the second best opinion-unware method. We believe that the PLCC gap is caused by the fact that the proposed method is not trained using Mean Opinion Score hence it is not forced to have a linear relationship with the target distribution. Moreover, as discussed in Section 3.3, SROCC is a more suitable metric than PLCC for the evaluation of ordinal variables.

In terms of correlation, DIIVINE appears to be the least correlated according to both SROCC and PLCC. ILNIQE and CORNIA are very close in terms of SROCC but they perform worse with respect to our method, while on the PLCC, CORNIA results to be the most effective method, followed by ILNIQE.

Regarding the capability of the methods to discriminate good quality images from the bad one, in terms of AUC, the CORNIA, and ILNIQE methods have similar performance, followed by the DIIVINE method which performs slightly better, and finally, by our method that achieves the best performance. Regarding the AUPR, the behavior is very similar to the AUC excepts for the DIIVINE method which results to be better than CORNIA.

### 4.2. Single Dataset Performance

The average results obtained on the three datasets show the generalization skills of the proposed algorithm. In Table 4 we report the performance of the methods under examination for each dataset.

On the LIVE-itW database the performance highlights that our method performs better in terms of SROCC with respect to all methods, while it is the best in terms of AUC and AUPR against all the opnion-unware methods but the second best (with a negligible difference of about 0.006 on average) with respect to the DIIVINE, which is an opinion-aware method. In terms of PLCC, ILNIQE is the best then followed by CORNIA, DIIVINE and our method. The LIVE-itW is the database on which all the methods perform worse with respect to the others datasets. This can be caused by the dimension of the dataset, which is one-tenth of the others.

On the KONIQ database, our method performs better in terms of SROCC and AUC with respect to all methods. Surprisingly, ILNIQE reaches the top performance on AUPR even if on the other measures is not highly competitive. The best in terms of PLCC is the CORNIA. Even in this case, performance of all methods, regardless the metric, are quite low.

Finally, on the SPAQ database, the overall behavior follows the one presented in the average performance (cf. Table 3): our method is the best in terms of SROCC, AUC, and AUPR while is not competitive in terms of PLCC.

Summing up, our method is first in terms or SROCC against all the methods over the three considered databases. It is the best opinion-unaware method in terms of AUC while it is the second best against an opinion-aware method only on the LIVE-itW dataset with a difference of 0.096. Since our method does not force any kind of linear relationship between the output and the data distribution, it is not competitive in terms of PLCC: it is the lowest performing method except for the SPAQ database were it is placed third. Considering the AUPR metric, except for the KONIQ database, we are the best of all methods on SPAQ and the second best on the LIVE-itW database of a small amount (0.0028).

**Figure 8:**
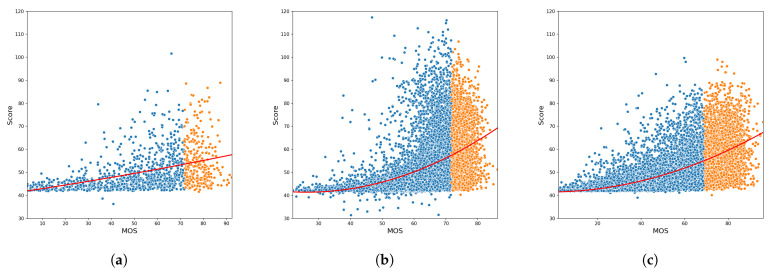
Scatter plots of the predicted quality scores respect MOS for the three considered datasets: (**a**) LIVE-itW, (**b**) KONIQ, and (**c**) SPAQ. In red is depicted the second-order interpolation line. The points in blue belongs to the bad quality images (MOS < 75° percentile of the MOS distribution for that dataset) while the orange ones refer to the good quality images.

**Figure 9:**
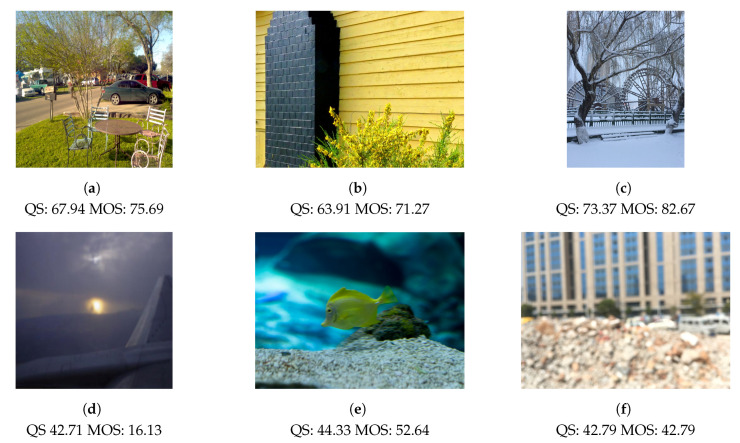
Examples of predicted quality score (QS) alongside the mean opinion score (MOS). First column images (**a**,**d**) belong to LIVE-itW database, second column picures (**b**,**e**) are from KONIQ dataset while the last column photos (**c**,**f**) belong to the SPAQ collection.

In Figure 8 we report, for each of the databases, the predicted quality score distributions with respect to the ground truth MOS. While in the Figure 9 are reported two pictures from the three dataset LIVE-itW, KONIQ, and SPAQ, alongside the predicted image quality scores and the mean opinion scores.

### 4.3. Ablation Study

In this section we focus on the contribution given by the anomaly detection method to the solely average of the intra-layer correlation. From Table 5 we can see that the intra-layer correlation achieves competitive results in terms of SROCC, AUC, and AUPR while is weaker with respect to the PLCC. On the other hand, the output from the anomaly detection method tends to be less effective on the four metrics. Although, the *abnormality score* tends to be more competitive on the PLCC except on the KONIQ database on which the PLCC scores is very low.

Even if the average intra-layer correlation alone shows interesting performance, combined with the *abnormality score* results in an increase of the PLCC while maintaining similar performance on the other metrics.

## 5. Conclusions

In this work, we have introduced a novel and completely blind image quality assessment method based on anomaly detection techniques. We have designed a model that classifies good and the bad quality pictures by exploiting the information deep encoded in CNN pre-trained on ImageNet, using only a subset of artifact-less images. To this end, we have developed a pipeline that relays on the Gram matrix computed over the activation volumes of a CNN to encode the intra-layer correlation. We then use this information in an anomaly detection fashion to improve the performance with respect to the only average of intra-layer correlation. Experimental results on three different datasets containing real distorted images demonstrated the effectiveness of the proposed approach. Moreover, cross-dataset results highlighted the robustness and generalization skills of the approach in comparison to other algorithms in the literature.

As future works, we would like to further investigate why the considered methods on certain databases, like SPAQ, perform on average better than on the others datasets. In view of the fact that our proposed algorithm lacks mainly concerning the PLCC, we are interested in improving such metric simultaneously with the others. To better compare our method with respect to the state of the art, we will take into account more recent approaches of no-reference opinion-unaware image quality assessment like the one by Mukherjee et al. [44]. Finally, to better prove the capability of the proposed method to discriminate good quality images with respect to bad quality ones, a possible alternative to the AUC and AUPR could be to take into account frameworks to stress image quality assessment methods [45].

## Figures and Tables

**Table 1:** Summary of the VGG16 [30] architecture alongside the resulting feature vector dimension with respect to the intra-layer correlation.

Block	Layer (Name)	Layer (Type)	Kernel Size	# Filters	Feature Vector Dimension
1	conv1_1	Convolutional	3×3	64	2016
	conv1_2	Convolutional	3×3	64	2016
	Max_Pooling	Pooling	-	-	-
2	conv2_1	Convolutional	3×3	128	8128
	conv2_2	Convolutional	3×3	128	8128
	Max_Pooling	Pooling	-	-	-
3	conv3_1	Convolutional	3×3	256	32,640
	conv3_2	Convolutional	3×3	256	32,640
	conv3_3	Convolutional	3×3	256	32,640
	Max_Pooling	Pooling	-	-	-
4	conv4_1	Convolutional	3×3	512	130,816
	conv4_2	Convolutional	3×3	512	130,816
	conv4_3	Convolutional	3×3	512	130,816
	Max_Pooling	Pooling	-	-	-
5	conv5_1	Convolutional	3×3	512	130,816
	conv5_2	Convolutional	3×3	512	130,816
	conv5_3	Convolutional	3×3	512	130,816
	Max_Pooling	Pooling	-	-	-
6	fc6	Dense			
7	fc7	Dense			

**Table 2:** Overview of databases used for image quality assessment with real distorted images.

Database	Year	No. of Images	Rating per Images	Environment	Inter-Group Agreement	MOS 75th Percentile
LIVE-itW [37]	2015	1162	175	crowd-sourcing	0.9896	71.74
KONIQ [39]	2018	10,073	120	crowd-sourcing	0.9730	71.71
SPAQ [38]	2020	11,125	15	lab	0.9230	68.82

**Table 3:** Pearson’s Linear Correlation Coefficient (PLCC), Spearman’s Rank-order Correlation Coefficient (SROCC) area under the receiver operating characteristic curve (AUC) and the area under the precision-recall curve (AUPR) of the proposed solution respect the three state of the art methods (ILNIQUE, CORNIA, DIIVINE). In each column, the best values are marked in boldface.

Method	SROCC	PLCC	AUC	AUPR
ILNIQUE ${}^{†}$ [23]	0.5596 ± 0.0000	0.5489 ± 0.0000	0.7327 ± 0.0000	0.4507 ± 0.0000
CORNIA * [17]	0.5638 ± 0.0000	**0.5859 ± 0.0000**	0.7329 ± 0.0000	0.4336 ± 0.0000
DIIVINE * [18]	0.5109 ± 0.0000	0.4870 ± 0.0000	0.7431 ± 0.0000	0.4501 ± 0.0000
Our ${}^{†}$	**0.5989 ± 0.0021**	0.4823 ± 0.0036	**0.7659 ± 0.0009**	**0.4710 ± 0.0006**

^†^ opinion-unaware methods, * opinion-aware methods.

**Table 4:** SROCC, PLCC, AUC, and AUPR of the proposed solution respect the three from state of the art methods (ILNIQUE, CORNIA, DIIVINE) for each datasets taken into account (LIVE-itW, KONIQ, and SPAQ). For each database, in each column, the best values are marked in boldface.

Evaluation Dataset	Method	SROCC	PLCC	AUC	AUPR
LIVE-itW	ILNIQUE ${}^{†}$	0.4516 ± 0.0000	**0.4968 ± 0.0000**	0.6610 ± 0.0000	0.3501 ± 0.0000
	CORNIA *	0.4307 ± 0.0000	0.4819 ± 0.0000	0.6598 ± 0.0000	0.3710 ± 0.0000
	DIIVINE *	0.4599 ± 0.0000	0.4504 ± 0.0000	**0.7127 ± 0.0000**	**0.4140 ± 0.0000**
	Our ${}^{†}$	**0.4933 ± 0.0018**	0.4058 ± 0.0028	0.7031 ± 0.0012	0.4112 ± 0.0007
KONIQ	ILNIQUE ${}^{†}$	0.5130 ± 0.0000	0.5026 ± 0.0000	0.7214 ± 0.0000	**0.4480 ± 0.0000**
	CORNIA *	0.5510 ± 0.0000	**0.5654 ± 0.0000**	0.7294 ± 0.0000	0.4218 ± 0.0000
	DIIVINE *	0.4734 ± 0.0000	0.4322 ± 0.0000	0.7177 ± 0.0000	0.4329 ± 0.0000
	Our ${}^{†}$	**0.5741 ± 0.0042**	0.4256 ± 0.0042	**0.7446 ± 0.0011**	0.4049 ± 0.0007
SPAQ	ILNIQUE ${}^{†}$	0.7142 ± 0.0000	0.6473 ± 0.0000	0.8156 ± 0.0000	0.5539 ± 0.0000
	CORNIA *	0.7096 ± 0.0000	**0.7103 ± 0.0000**	0.8094 ± 0.0000	0.5080 ± 0.0000
	DIIVINE *	0.5993 ± 0.0000	0.5784 ± 0.0000	0.7989 ± 0.0000	0.5035 ± 0.0000
	Our ${}^{†}$	**0.7292 ± 0.0002**	0.6155 ± 0.0038	**0.8501 ± 0.0003**	**0.5970 ± 0.0003**

${}^{†}$ opinion-unaware methods, * opinion-aware methods.

**Table 5:** SROCC, PLCC, AUC and AUPR of the proposed solution alongside the average intra-layer correlation (MEAN_GM) and the *abnormality score* for the databases LIVE-itW, KONIQ and SPAQ. For each database, in each column, the best values are marked in boldface.

Dataset	Method	SROCC	PLCC	AUC	AUPR
LIVE-itW	MEAN_GM	0.4930 ± 0.0000	0.3520 ± 0.0000	**0.7043 ± 0.0000**	0.4068 ± 0.0000
	ABNORMALITY SCORE	0.4599 ± 0.0075	0.3609 ± 0.0266	0.6826 ± 0.0045	0.3966 ± 0.0035
	Our	**0.4933 ± 0.0018**	**0.4058 ± 0.0028**	0.7031 ± 0.0012	**0.4112 ± 0.0007**
KONIQ	MEAN_GM	0.5512 ± 0.0000	0.3050 ± 0.0000	0.7379 ± 0.0000	0.3998 ± 0.0000
	ABNORMALITY SCORE	0.4873 ± 0.0220	0.1343 ± 0.0335	0.7169 ± 0.0102	**0.4380 ± 0.0082**
	Our	**0.5741 ± 0.0042**	**0.4256 ± 0.0042**	**0.7446 ± 0.0011**	0.4049 ± 0.0007
SPAQ	MEAN_GM	0.7281 ± 0.0000	0.5227 ± 0.0000	**0.8505 ± 0.0000**	0.5934 ± 0.0000
	ABNORMALITY SCORE	0.6590 ± 0.0152	0.4387 ± 0.0526	0.8037 ± 0.0104	0.5525 ± 0.0100
	Our	**0.7292 ± 0.0002**	**0.6155 ± 0.0038**	0.8501 ± 0.0003	**0.5970 ± 0.0003**
AVERAGE	MEAN_GM	0.5908 ± 0.0000	0.3932 ± 0.0000	0.7642 ± 0.0000	0.4667 ± 0.0000
	ABNORMALITY SCORE	0.5354 ± 0.0149	0.3113 ± 0.0376	0.7344 ± 0.0084	0.4624 ± 0.0072
	Our	**0.5989 ± 0.0021**	**0.4823 ± 0.0036**	**0.7659 ± 0.0009**	**0.4710 ± 0.0006**

## Data Availability

Not applicable.
